# A Focused Review of Gamma Neuromodulation as a Therapeutic Target in Alzheimer’s Spectrum Disorders

**DOI:** 10.20900/jpbs.20240001

**Published:** 2024-02-28

**Authors:** I-Wei Shu, Yayu Lin, Eric L. Granholm, Fiza Singh

**Affiliations:** 1 Department of Psychiatry, University of California San Diego, 9500 Gilman Drive, La Jolla, CA 92093, USA; 2 Swartz Center for Computational Neuroscience, Institute for Neural Computation, University of California San Diego, La Jolla, CA 92093, USA; 3 Department of Electrical and Computer Engineering, University of California San Diego, La Jolla, CA 92093, USA

**Keywords:** mild cognitive impairment, gamma oscillations, transcranial alternating current stimulation, transcranial direct current stimulation, EEG neurofeedback

## Abstract

The aging population of the world is increasing at an unprecedented rate which is expected to lead to a corresponding unparalleled increase in age related diseases. Of particular concern are the large number of older adults expected to develop Alzheimer’s disease (AD), which will require extraordinary local, national and worldwide healthcare resources. In this context, innovative interventions are needed urgently to delay AD onset and thereby give our healthcare systems time to prepare and provide meaningful care to our aging populations. This focused review discusses the crucial role of frontal gamma oscillations as a therapeutic target to delay or ameliorate cognitive decline in AD. Frontal gamma oscillations, including from prefrontal cortical areas, serve as a biomarker for working memory and other cognitive functions, and their impairment is observed before clinical symptoms manifest. This review evaluates evidence from animal models and human subjects to highlight the correlation between gamma wave abnormalities and cognitive deterioration. Furthermore, the review summarizes 11 clinical studies using neuromodulation techniques designed to stimulate gamma oscillations in mild cognitive impairment (MCI) and AD patients, including transcranial electrical stimulation, transcranial magnetic stimulation, and rhythmic sensory stimulation. These interventions have shown promise in mitigating early-stage cognitive decline, as evidenced by improved performance on memory tests, increased gamma oscillatory responses, and some have even shown reduced brain atrophy. These early studies suggest that treatments that strengthen frontal gamma oscillatory responses through neuromodulation are a promising approach to delay cognitive decline, that may serve as an adjunct to other therapies or as a standalone treatment in some populations.

## INTRODUCTION

The last 50 years of medical advances have led to dramatic increases in lifespan such that, by the year 2050, over 2 billion people worldwide will be over 60 years of age [1]. Among them, more than 130 million will develop some form of dementia, with Alzheimer’s Disease (AD) being the most common [2,3]. In the United States (US) alone, where AD currently affects over 6 million older adults [1], prevalence is expected to more than double by 2050 and reach a staggering 13 million, the equivalent of 1 in 25 Americans. Thus, interventions that delay the onset of dementia are urgently needed to both improve the wellness or functional lifespan of our aging population and reduce caregiver and societal costs to realistic and manageable levels. According to one study, with over 900,000 new AD cases in the US every year, the ability to delay illness onset by 5 years now would reduce the total number of cases by over 3 million, and total healthcare costs by over 33%, by 2050 [4]. Without new treatment options, total economic costs of treating and caring for AD (currently estimated at over $600 billion, i.e., over $350 billion in direct healthcare costs plus over $250 billion in unpaid caregiver costs) is expected to approach $2 trillion by 2050 [1].

Therefore, treatments that delay the onset of disease are urgently needed to mitigate oncoming economic and other costs. In this context, mild cognitive impairment (MCI) and subjective cognitive decline (SCD), two high risk states that increase the risk of developing AD are especially noteworthy timepoints for early intervention. The diagnosis of MCI consists of cognitive impairments that can be readily demonstrated with behavioral tests, but where the individual does not exhibit significantly impaired levels of function. A diagnosis of MCI leads to a 10-fold increase in the risk for developing AD compared to age matched controls. Patients meeting criteria for SCD also exhibit increased risk for developing AD (though risk is less, when compared to patients with MCI) [5–8]; more specifically, among the 15 million older Americans meeting SCD criteria, 40 percent will progress to some form of dementia within 20 years.

Despite widespread awareness of the high personal and socio-economic costs to individuals and communities, there are few treatment options for AD disorders. Medications approved in previous decades (cholinesterase inhibitors, memantine) offer only modest effects on cognition and do not alter the course of illness [2,10]. Newer anti-amyloid antibody infusions (e.g., aducanumab, lecanemab) offer promise; however, these medications come with serious side-effects and risks, as well as extraordinary costs ($10,000 to $37,000 per patient per year), which will limit their access to millions of older adults in the early stages of cognitive decline. Therefore, these factors underscore the urgent need for novel approaches to produce cost-effective and accessible interventions for older adults with emerging cognitive decline.

Within the past decade, external stimulation of high-frequency (e.g., gamma) neural activity has emerged as a promising approach towards attenuating or reversing AD-related declines in cognitive and neurologic health [11–14]. Building on the information presented in these reviews, we detail here the biological relationship between gamma activity and information processing, and how this biology presents frontal gamma responses, especially in the context of working memory (WM), as an especially promising treatment target for patients at risk for, or exhibiting, AD-related cognitive decline. We revisit gamma neuromodulation studies targeting AD-related cognitive decline, with a special focus on their lessons for our proposed WM-informed approach towards frontal gamma neuromodulation. We conclude by reviewing how electroencephalographic (EEG) neurofeedback (NFB) targeting frontal gamma activity may provide unique advantages and information for this emerging landscape of accessible and promising treatment options, which directly engage neural function to improve cognitive health for patients at risk for, or exhibiting, AD-related difficulties.

## NEURAL BASIS OF WORKING MEMORY AND OTHER COGNITIVE FUNCTIONS

Our ability to perform complex, goal-directed behaviors requires coordinated activity across multiple brain regions including frontal, parietal and limbic/paralimbic (e.g., cingulate) brain areas [15]. Synchronous, oscillatory neural activity that arises from activation of large neuronal assemblies across these brain areas support a set of closely-related cognitive processes (e.g., executive functions) including sustained attention, planning, cognitive control and working memory (WM). WM consists of our mental ability to internally maintain and manipulate task-relevant information [15,16], and is especially relevant for everyday functioning. In coordination with related cognitive processes, WM supports complex behaviors in both humans and animal models, and is significantly impaired in patients with neuropsychiatric disorders, e.g., schizophrenia, major depression, posttraumatic stress disorder and AD [17,18].

During tasks assessing WM and related cognitive functions, task-relevant brain areas generally exhibit increased high-frequency, or gamma (e.g., 30–50 Hz), neural activity, in an event specific manner, supporting gamma synchronization as a conserved mechanism for information processing by neural assemblies [19].

For example, increasing intensity of external visual stimuli (relative to background) increases occipital-parietal gamma activity [20]. Similarly, recognition of internally-maintained visual information (relative to distractor stimuli) increases occipital-parietal gamma activity [21]. In contrast to gamma activation of task-related cortical areas, lower-frequency oscillations, e.g., alpha (8–14 Hz) or beta (15–30 Hz), generally inhibit, or otherwise modulate, task-irrelevant areas [22]. Beta activation is most famously observed at corresponding cortical sensorimotor areas during inhibition of motor movements, i.e., “beta inhibition” [23]. Conversely, central-posterior gamma synchronization during visual recognition is accompanied by central-posterior beta desynchronization i.e., “beta inhibition” being turned off [21,24]. Consistent with this model, during facial recognition, posterior gamma synchronization is similarly accompanied by posterior alpha and beta desynchronization [25]. As expected, these general responses during cortical processing of sensory stimuli become more varied during more complex information processing tasks. For example, when instructed to “encode” visual stimuli during WM tasks, target visual stimuli can produce theta and alpha (though not generally beta), in addition to gamma, synchronization [21,25,26]. During the “retention” or “maintenance” intervals of such tasks, theta and alpha synchronizations generally diminish, with gamma synchronization generally being preserved. Opposing interactions between beta and gamma synchronization can be observed during this interval, likely indications of internal representations being manipulated. Responses during the “recall” or “retrieval” phase of these tasks generally mirror responses during the “encoding” phase.

During the “retention” or “maintenance” intervals of WM tasks, frontal gamma synchronization may be a relatively specific marker of prefrontal gamma activity. In support of this model, Semprini and colleagues demonstrated that the maintenance phase of the N-Back (a WM task) was associated with significant gamma synchronization, especially in frontal, and dorsal-lateral prefrontal (DLPFC) areas [27]; in contrast, gamma activity during the encoding and retrieval phases were relatively attenuated (with theta and beta activity being more prominent). Tallon-Baudry and colleagues similarly demonstrated significant frontal (e.g., F3, F4) gamma synchronization during the maintenance interval of a visual WM task (delayed match to sample, or DMS), compared to control intervals where maintenance were not required [21]. In another study assessing WM function using a visual DMS task, Honkanen and colleagues also demonstrated that only gamma activity was significantly increased in prefrontal areas during the maintenance phase (in contrast to other frequencies, e.g., beta) [28]. A specific role for DLPFC gamma synchronization during WM was further supported by Roux and colleagues demonstrating that, during a DMS task with varying loads (0, 3, or 6 items), only medial and DL PFC (i.e., Brodmann Area 9) gamma activity correlated with and predicted performance in a load-dependent manner [29].

The potential for frontal gamma synchronization to serve as a relatively specific marker of prefrontal gamma activity is further supported by the routine use of frontal EEG electrodes (i.e., F3, F4) as locations for assessing or targeting DLPFC activity, e.g., during near-infrared spectroscopy, transcranial magnetic stimulation (TMS), respectively. Anatomically, the electrode sites F3 and F4 (from the International 10–20 System for EEG electrode placement) are approximately 14 mm directly above the left and right DLPFC in 81% and 98% of individuals, respectively, with standard deviation +/− 8 mm [30]. The limited simultaneous intracranial electrocorticography (ECoG) and EEG studies available have also reported high correlation between DLPFC and F3/F4 neurophysiologic activity [31]. Consistent with this model, in a simultaneous TACS and functional magnetic resonance imaging (fMRI) study of 15 healthy volunteers, Mencarelli and colleagues demonstrate that 16 min of gamma-TACS (F3, anode; F4, cathode; 40 Hz; 2 mA; 60 s on; 60 s off), produced increased DLPFC blood oxygen level dependent (BOLD) fMRI signal (with secondary activation of cingulate, motor, temporal and visual areas) [32]. Similarly, in a simultaneous TMS/TACS study of 13 healthy volunteers, Maiella and colleagues demonstrated that intermittent theta burst TMS stimulation (iTBS), which involves bursts of gamma (50 Hz) stimulation at rates equivalent to theta (e.g., bursts every 200 ms being equivalent to 5 Hz), combined with gamma-TACS (F3 or F4, anode; R deltoid muscle, cathode; 70 Hz, 1 mA; synchronized to iTBS) produced greater gamma responses (compared to theta- or sham-TACS) at DLPFC sources (localized using BrainVision) receiving stimulation, and not contralateral DLPFC or other sources [33]. Thus, despite the limited anatomic resolution of EEG in general, frontal gamma activity, especially when recorded at F3 and F4 during WM tasks, may be a relatively specific marker of DLPFC activation.

## GAMMA WAVE ABNORMALITIES IN AD

Consistent with gamma synchronization’s critical role in optimal memory function, the role of abnormal gamma oscillations in AD has been observed since the early 1990’s, for example, in an early case report (from 1991) of decreased global gamma MEG activity in patients with Alzheimer’s [34], as well as in more recent studies [35–38]. More specifically, compared to matched controls, older adults with amnestic MCI exhibit decreased gamma activity, which correlated with decreased verbal learning performance [39]. Interestingly, even in the absence of frank cognitive impairment, older adults with abnormal amyloid and tau levels exhibit decreased gamma activity and WM performance, compared to matched controls with normal amyloid/tau levels [40]. Some studies have reported ambiguous results or no differences in gamma activity between AD patients and healthy control participants; however, gamma activity was in fact not analyzed (e.g., technical difficulties with artifact removal) in several such studies [41,42]. In their review of conflicting evidence, Babiloni and colleagues suggest that the “relatively low sampling frequency” utilized in many studies precludes specific assessment of “EEG signal beyond 40 Hz” [43].

Specific assessments of frontal gamma responses (within the context of the model presented in the previous section) has been further confounded by methodologic and pathophysiologic variability in these studies. For example, studies demonstrating lower levels of gamma functionality generally utilize measures of cross-electrode or cross-frequency coupling [38,44–47]. In studies utilizing procedures mirroring those discussed above, the healthy comparison groups generally have not exhibited expected event-related gamma responses; therefore, any deviations exhibited by the AD groups are not easily reconciled with established neural models of cognitive function [36,48,49]. Conversely, in one study where healthy controls (*n* = 27) did, in fact, exhibit expected frontal (Fz) gamma synchronization during the N-Back, participants with MCI (*n* = 21) and AD (*n* = 16) exhibited > 25% decrease in gamma synchronization in comparison [50]. In another study utilizing the N-Back, compared to stable MCI patients (*n* = 13), MCI patients with cognitive decline one year following baseline assessment (*n* = 16) exhibited decreased frontal gamma responses during baseline N-Back assessment [51]. Methodologic variability commonly contribute to described variability among results across studies; for example, we have observed, in studies of WM-related frontal gamma responses in patients with schizophrenia, extracting gamma power over time windows on the order of 100–500 ms after stimulus onset (a time window mostly devoted to encoding and retrieval during WM tasks) can produce effects opposite to time windows more specifically aligned with retention/maintenance during WM tasks [52].

Specific to studies of patients at risk of, or with, AD, Gaubert and colleagues have also demonstrated that pathophysiologic status (e.g., amyloid levels) can produce opposite effects on gamma activity [53]. Initially, amyloid decreases gamma activity. Further accumulation then generally increases gamma activity until, at the highest levels of amyloid, gamma activity again decreases. Consistent with abnormal amyloid disrupting frontal gamma synchronization, compared to older adults with normal cerebrospinal amyloid levels, older adults with abnormal cerebrospinal amyloid levels exhibit lower N-Back accuracy and frontal gamma synchronization [40]. In a recent effort to more mechanistically interrogate frontal gamma activation in AD patients, Casula and colleagues demonstrated that, compared to 21 healthy controls, 60 participants with AD exhibited significantly lower DLPFC gamma responses to transcranial magnetic stimulation (TMS, *t* = −2.977, *p* = 0.004) [54]. Furthermore, DLPFC gamma responses significantly predicted AD status (in a regression model), positively-correlated with cortical plasticity (measured by theta burst induced changes in motor evoked potentials) and negatively-correlated with CSF tau (including phosphorylated tau, but not amyloid).

## GAMMA WAVE ACTIVATION AS A THERAPEUTIC TARGET IN AD

Despite heterogeneity across studies, that patients at risk of, or with, AD generally exhibit disturbed gamma responses has led to studies testing gamma neuromodulation as a potential treatment for AD-related cognitive decline. Prior to clinical availability of non-invasive neuromodulation, deep brain stimulation (DBS) was tested to increase gamma synchronization in AD patients with promising results. For example, in a case series of 6 participants with mild to moderate AD, Laxton and colleagues demonstrated that 12 months of chronic bilateral deep-brain fornix stimulation was associated with improved cognitive and behavioral measures in most patients [55]. Furthermore, results from mechanistic inquiries in this study indicated that bilateral deep-brain fornix 130 Hz stimulation activates bilateral temporal (including hippocampal), cingulate (including anterior cingulate) and medial prefrontal activity in the near-term; and, additional parietal and prefrontal areas in the long term. Similarly, in a case series of 7 participants with intracranial depth electrodes for seizure evaluation, Suthana and colleagues demonstrated that entorhinal (but not hippocampal) 50–130 Hz stimulation improved visual-spatial memory [56]. In this same study, mechanistically, entorhinal stimulation was associated with hippocampal theta phase resetting.

More recently, enhancing gamma activation with sensory and electromagnetic approaches has demonstrated promise for AD-related cognitive decline [57,58]. The non-invasive neuromodulation therapies proven to be safe and efficient for treating brain disorders include several modalities. These modalities include rhythmic sensory stimulation (RSS), which utilizes auditory and visual stimulation; transcranial alternating (TACS) or direct current stimulation (TDCS), which modulate cortical activity with low-intensity currents; TMS, which induces cortical currents electromagnetically; and neurofeedback (NFB), which utilizes self-regulated neural responses. For example, older adults with early AD receiving gamma rhythmic sensory stimulation (RSS) exhibit improved memory function and reduced loss of brain volume, compared to participants with early AD receiving placebo/control intervention [57]. Another study of gamma RSS on 10 MCI patients showed enhanced functional connectivity after 8 weeks of training [59].

With specific regards to modulating gamma activity with visual stimuli, Duecker and colleagues demonstrated that gamma responses to high-frequency flicker may be independent of, and exhibit minimal interaction with, gamma responses to dynamic visual gratings [60]. More specifically, while visual flicker at varying frequencies specifically produced gamma synchronization at corresponding frequencies (from MEG sources projecting to occipital areas), when simultaneously presenting flicker and dynamic grating stimuli, grating-related gamma responses were not further focused by frequency of flicker stimuli. In contrast to the absence of interaction between flicker- and grating-related gamma responses, Lobo and colleagues observed partial but significant interactions between flicker- and grating-related gamma responses in over one-third of participants in a similarly designed MEG study primarily focused on gamma responses to 60 Hz flicker [61]. Lobo and colleagues further report that, in Duecker and colleagues’ report, a similar interaction is visible in an early and primarily methods-focused figure with data from individual participants, indicating that heterogeneous results across studies may arise form methodologic variability, especially given features unique to gamma responses (e.g., high frequency responses of low power). In addition to these and other methodologic differences (e.g., stimulus design, MEG feature extraction), Lobo and colleagues’ study also specifically required participants to attend to active stimuli, whereas participants in Duecker and colleagues’ study were instructed to attend to distractor stimuli, consistent with attention and other cognitive events being important modulators of gamma responses. For example, in a study requiring participants to attend (or ignore) active (or inactive) unilateral flicker stimuli (a design enabling isolation of effects from attention, inattention, flicker on, flicker off, and hemisphere) Gulbinaite and colleagues demonstrate that flicker produced synchronous interactions between flicker-related and endogenous occipital gamma responses, both generally and at specific flicker frequencies [62]. Compared to ignore trials, attend trials further enhanced synchronous gamma interactions; in contrast, consistent with model presented above, synchronous alpha interactions were greater during ignore trials (alpha suppression of task-irrelevant areas). Of note, given gamma’s relatively low power, and sensitivity to methodologic variability, Gublinaite and colleagues developed a custom feature extraction approach (from established source separation and clustering methods) specifically for visual flicker stimuli [63], which was utilized for both alpha and gamma responses in this study.

While not a specific test of synchronous interactions between stimulus-related and endogenous gamma responses, of neurophysiologic and methodologic interest, Wang and colleagues (by utilizing a WM design where visual stimuli flickered at, and volume of auditory stimuli pulsed at, gamma frequencies) demonstrated that synchronous interactions between gamma responses to separate stimuli can be modulated by varying relative phase of external stimuli [64]; a follow-up analysis further demonstrated modulation by optimal memory performance, i.e., greater synchronous interactions between gamma responses to visual and auditory stimuli (including of prefrontal and hippocampal gamma responses) during correct trials [65]. This preprint by Kahn and colleagues further discusses similarities and differences between studies by Tsai and colleagues and Soula and colleagues [66], with specific regards to investigating gamma sensory stimulation in animal models of AD beyond the scope of this review.

## RECENT STUDIES TESTING GAMMA NEUROMODULATION AS TREATMENT FOR AD

To further explore evidence for gamma neuromodulation as a treatment option for patients with AD, we searched PubMed for reports of gamma (or 40 Hz) neuromodulation (e.g., transcranial magnetic or electrical stimulation) for patients at risk of, or with, AD (Table 1). Eight studies were not included in Table 1 due to (1) including non-AD forms of dementia [67], (2) providing descriptive statistics only [68,69], (3) statistics not directly controlling for control condition, i.e., within-group statistics only [58,59,70,71], or (4) testing novel TMS device for unsupervised patient use at home, without providing generally required adherence and fidelity information [72]. Please also see review by McDermott and colleagues for review of older reports, most not included in PubMed [73].

Supporting McDermott and colleague’s conclusion in 2018 that “the 40 Hz frequency value seems of particular neurological importance and as such represents a natural target value [with] promise for clinical application to AD”, the studies presented Table 1 establish gamma neuromodulation as a promising treatment option for patients at risk of, or with, AD. As the approach tested in 6 studies, TACS currently provides the most evidence supporting a therapeutic role for gamma neuromodulation. Consistent with the model presented above, half of the included TACS studies targeted frontal areas. More specifically, Kim and colleagues demonstrated that a single 30 min session of 40 Hz TACS stimulation at F3 or F4 (i.e., bilateral DLPFC) improved cognitive control (as measured by Stroop performance) and trail making (a test of executive function with emphasis on attention, processing speed and flexibility) in participants with MCI [75]. Administering daily sessions of frontal gamma TACS weekly, over 2–4 weeks, was further associated with increased bilateral hippocampal perfusion and bilateral temporal gamma activity [77]; and, simultaneous administration with a memory challenge for 8 sessions over 4 weeks was associated with improved WM and verbal fluency [81]. In addition to frontal areas, gamma TACS stimulation at Pz (e.g., precuneus) and of the angular gyrus, whether a single session or daily sessions over weeks, have also been associated with improved WM and verbal fluency [82], including in comparison to sham stimulation [74,78].

While significantly better established clinically, only two studies utilized TMS, though both demonstrated a positive effect of multiple TMS sessions per week, over 2–6 weeks, targeting DLPFC/parietal areas, or precuneus, on coupling of gamma activity across distributed cortical networks, WM and trail making [80,83]. That TACS, despite being much less well established clinically, exhibits greater popularity than TMS likely arises from TACS being significantly more cost-effective, portable and accessible [12]. Furthermore, while rare, TMS nevertheless exhibits significantly higher seizure risk than TACS, a potentially serious risk in older patients with neurologic decline [85,86].

The remaining studies included in Table 1 utilize gamma RSS. While gamma RSS had been well established as a method for stimulating gamma responses [87,88], Iaccarino and colleagues first established the therapeutic potential of gamma RSS for patients at risk of, or with, AD by demonstrating that both internal (using optogenetic) and external (visual flicker) gamma stimulation reduces amyloid-beta levels in a mouse model of AD, most likely by increasing gamma and glial responses [89]. These results aligned well with existing studies associating disturbed gamma activity and amyloid-beta levels in both mouse models of and patients with AD [90]. More recently, testing in patients at risk of, or with, AD have confirmed gamma RSS as a promising treatment option (Table 1). More specifically, in 3 sham-controlled studies (*n* = 47 total active participants; 28, control condition), simultaneous gamma auditory-visual RSS was associated with reduced overnight restlessness, loss of daily function, ventricular enlargement, and loss of CNS white matter, as well as improved semantic memory performance [76,79,84].

To summarize Table 1, the 11 included studies represent a sample of *n* = 214 participants at risk of, or with, AD exhibiting improved cognitive (e.g., executive, attention, processing speed, WM, semantic memory, verbal fluency), clinical (e.g., sleep, daily), and neurophysiologic (e.g., gamma) function, as well as improved bilateral hippocampal perfusion, and reduced ventricular enlargement, and loss of CNS white matter (in comparison to *n* = 165 control participants in 6, or just over half, of the studies). For clarity and conciseness, we included in Table 1 only select outcome measures (those most relevant to the models of AD pathophysiology presented above). While not all positive and negative outcome measures were presented, the included outcome measures were accepted for publication, and we defer to the publishers that data analytic methods (e.g., multiple comparisons corrections) were valid. Of the eight studies not included in Table 1 (see above rationale), 7 reported similarly positive outcome measures [58,59,67–71], and only 1 reported only negative outcome measures [72]. As mentioned above, we did not include the study reporting only negative outcomes in Table 1 due to the study not reporting adherence and fidelity information generally required for the testing of a novel TMS device for unsupervised patient use at home.

Motivated by these promising results, we have developed an additional approach towards enhancing frontal gamma activity using EEG neurofeedback (NFB). Briefly, EEG-NFB is a form of operant conditioning where an EEG feature is coupled to, generally visual and/or auditory, positive and negative reinforcement signals; for example, sound or music, slideshows, digital games [91]. In particular and in light of findings discussed above, enhancement of gamma activity is expected to improve WM and other cognitive functions for patients at risk of or with AD. Support for this hypothesis comes from the findings that gamma-NFB, but not alpha-, beta-, or placebo-NFB, is associated with improvements in visual processing and memory [92,93]. NFB inherently offers advantages in that training can be easily (1) personalized (e.g., personalized media/games); and, (2) may be used to target additional EEG features. As a first step forward, we reported last year preliminary results from a double-blind, placebo-controlled clinical trial of gamma NFB (30 min, 2/week, 12 weeks) for patients with MCI (*n* = 9 active; 9, placebo) [94]. More specifically, we demonstrate that, compared to placebo-NFB, patients receiving active-NFB exhibit significantly increased frontal gamma responses during training. In this ongoing study, early data suggest that in those undergoing gamma-NFB, baseline F4 gamma power (but not at other electrodes) is significantly correlated with the slope of training-related increases in frontal gamma responses – consistent with frontal gamma responses being an important neural event and promising therapeutic target for understanding and treating AD-related cognitive decline.

## SUMMARY AND FUTURE DIRECTIONS

The “silver tsunami” of aging adults is on its way, and a thoughtful, concerted response is needed from our healthcare system to ensure timely and comprehensive care for our aging adults. Although medical advances have increased lifespans, a corresponding increase in the wellness span of aging adults has not been realized. Furthermore, statistical models predict an unprecedented increase in the number of older adults with cognitive impairment and AD disorders, requiring enormous local, national and global resources by 2050. Thus, cost- and resource-efficient solutions to slow down cognitive aging are needed NOW. In this regard, recent advances in our understanding of the neural basis of short-term memory, especially WM, coupled with emergent neuromodulation techniques present opportunities for development of novel interventions to slow down cognitive decline. The field is in its nascent stage and although the scientific rationale is well delineated, few clinical studies have been conducted to date. Results from a handful of studies using TMS, tACS, tDCS, RSS and EEG-NFB are notable in that these modalities appear feasible, are largely well tolerated and show early indications of separation from placebo. Furthermore, promising results from this growing body of literature suggest that frontal neural circuitry can be engaged using these neuromodulation techniques in individuals in early stages of cognitive decline. Fortuitously, engaging this circuitry is associated with changes at virtually every level including brain substance, neurophysiology and behavior, making a compelling case for the mechanistic role of this circuitry in disorders of cognition. Additional studies are needed to further delineate dose-response curves, safety profiles and rational combinations of these promising treatments with other modalities.

More specifically, each of the modalities offers some specific advantages and disadvantages. For instance, TMS has been tested and FDA approved for other indications including depression and is therefore the farthest along in terms of clinical application; however, TMS requires significant investment in equipment and staff, can produce distress for some patients, and is associated with very low but nevertheless concerning risk for seizures. TACS has a lower resource burden, but at present the safety profile of current stimulation protocols remains scant. RSS is an inexpensive and low resource requiring modality; however, it is also one of the newest and clinical applications are several years away. EEG-NFB is unique in that it trains the brain’s intrinsic mechanisms and optimizes neural processing without external stimulation and therefore may be an option as a standalone or adjunctive treatment with other therapies. An added advantage of EEG-NFB comes from the fact that its safety profile is well established, and with the advent of improved hardware and software, EEG-NFB can be delivered with much greater specificity than previously possible. Nonetheless, EEG-NFB also presents some challenges in that it relies on effort and would therefore be primarily suitable for motivated individuals. Despite some limitations, in large part, direct brain treatments present an emerging, and desperately needed area of therapeutic development for aging adults at risk of developing AD and related dementias. These treatments can be implemented alone or as dose-lowering strategies or adjuncts to other more invasive and costly treatments. We urge the field to take a thoughtful and open-minded approach to respond to the emerging public health crisis of AD and related dementias.

## Figures and Tables

**Table 1.** Summary of gamma neuromodulation studies on MCI or AD patients.

Reference	Modality	Frequency, Location	Participants	Protocol	Select Outcome Measures	Results
Benussi et al, 2021 [74]	TACS	40 Hz, 60 min, Pz	MCI (*n* = 20, active/sham cross-over)	1 session	WM, semantic memory	Compared to control condition, 40 Hz TACS associated with improved WM and semantic memory performance
Kim et al, 2021 [75]	TACS	40 Hz, 30 min (bilateral DLPFC, i.e., F3, F4)	MCI (*n* = 20, TACS/TDCS/sham crossover)	1 session	Cognitive control (Stroop), trail making	Compared to TDCS, 40 Hz TACS improved sham, tACS significantly improved Stroop and trail making performance; compared sham, 40 Hz TACS significantly improved trail making performance
Cimenser et al, 2021 [76]	RSS	40 Hz simultaneous auditory-visual RSS	AD (*n* = 14, active; 8, sham)	1 hour daily x 6 months	Sleep actigraphy, daily level of function	Compared to sham, 40 Hz RSS associated with significantly less restlessness overnight and loss of daily function
Sprugnoli et al, 2021 [77]	TACS	40 Hz, 60 min (frontal/temporal)	AD (*n* = 15, active; no control condition)	5 daily sessions per week × 2 to 4 weeks	MRI: cerebral perfusion (ASL), resting gamma EEG activity	Increased bilateral hippocampal perfusion and bilateral temporal gamma activity
Benussi et al, 2022 [78]	TACS	40 Hz, 60 min, Pz	AD (*n* = 60, active/sham cross-over)	1 session	WM, semantic memory	Compared to control condition, 40 Hz TACS associated with improved WM and semantic memory performance
Chan et al, 2022 [79]	RSS	40 Hz simultaneous auditory-visual RSS	AD (*n* = 8, active; 7, control condition)	1 h daily × 3 months	Structural MRI, semantic memory	Compared to control condition, 40 Hz RSS associated with significantly less ventricular enlargement, and improved semantic memory performance
Traikapi et al, 2023 [80]	TMS	40 Hz, ~45 min (L/R precuneus)	AD (*n* = 4, active; no control condition)	10 sessions over 2 weeks	WM, trail making	1 patient exhibited significant WM and trail making improvements; a second patient exhibited significant trail making improvements
Jones et al, 2023 [81]	TACS	40 Hz, 60 min (anterior frontal/temporal/parietal) administered with memory challenge	MCI (*n* = 13, active; no control condition)	8 sessions over 4 weeks	CVLT, verbal fluency	Improved WM subscore of CVLT; improved verbal fluency
Cappon et al, 2023 [82]	TACS	40 Hz, 20 min (L angular gyrus)	AD (*n* = 8, active; no control condition)	5–7 sessions per week × 14 weeks	MoCA	Improved WM subscore of MoCA; no change in MoCA
Hoy et al, 2023 [83]	TMS	50 Hz iTBS (L/R DLPFC, L/R parietal)	AD (*n* = 29, active; 27, sham)	21 sessions over 6 weeks	Distributed gamma coupling, WM	Compared to sham, active 50 Hz iTBS associated with increased gamma coupling and WM function
Da et al, 2024 [84]	RSS	40 Hz simultaneous auditory-visual RSS	AD (*n* = 25, active; 13, sham)	1 h daily × 6 months	Structural MRI: CNS white matter	Compared to sham, 40 Hz RSS associated with significantly less white matter loss

## Data Availability

This is a review article, and no data were generated from the study.

## ABBREVIATIONS

Aβ: beta amyloid
AD: Alzheimer’s Disease
CNS: central nervous system
DBS: deep brain stimulation
DLPFC: dorsolateral prefrontal cortex
DMPFC: dorsomedial prefrontal cortex
EEG: electroencephalography
MCI: mild cognitive impairment
MRI: magnetic resonance imaging
NFB: neurofeedback
RSS: rhythmic sensory stimulation
SCD: subjective cognitive decline
TACS: transcranial alternating current stimulation
TDCS: transcranial direct current stimulation
TMS: transcranial magnetic stimulation
US: United States
WM: working memory
